# Association of Timing of Adjuvant Therapy With Survival in Patients With Resected Stage I to II Pancreatic Cancer

**DOI:** 10.1001/jamanetworkopen.2019.9126

**Published:** 2019-08-14

**Authors:** Sung Jun Ma, Oluwadamilola T. Oladeru, Joseph A. Miccio, Austin J. Iovoli, Gregory M. Hermann, Anurag K. Singh

**Affiliations:** 1 Department of Radiation Medicine, Roswell Park Comprehensive Cancer Center, Buffalo, New York; 2 Department of Radiation Oncology, Massachusetts General Hospital, Boston, Massachusetts; 3 Department of Therapeutic Radiology, Yale University School of Medicine, New Haven, Connecticut; 4 Jacobs School of Medicine and Biomedical Sciences, University at Buffalo-State University of New York (SUNY), Buffalo

## Abstract

**Importance:**

Surgery followed by adjuvant chemotherapy or chemoradiation is widely used to treat resectable pancreatic cancer. Although studies suggest initiation of adjuvant therapy within 12 weeks of surgery, there is no clear time interval associated with better survival.

**Objective:**

To evaluate the ideal timing of adjuvant therapy for patients with stage I to II resected pancreatic cancer.

**Design, Setting, and Participants:**

This cohort study included 7548 patients with stage I to II resected pancreatic cancer (5453 with adjuvant therapy; 2095 without adjuvant therapy) from the National Cancer Database from 2004 to 2015. Data were collected from January 2014 to December 2015 and analyzed from December 2018 to May 2019.

**Exposures:**

Adjuvant chemotherapy or chemoradiation at various time intervals.

**Main Outcomes and Measures:**

Overall survival (OS).

**Results:**

A total of 7548 patients (3770 male [49.9%]; median [interquartile range] age, 67 [59-74] years) were identified from the National Cancer Database. Among 5453 patients with adjuvant therapy, a Cox model with restricted cubic splines identified the lowest mortality risk when adjuvant therapy was started 28 to 59 days after surgery. Patients were divided into early (n = 269, adjuvant therapy initiated within <28 days), reference (n = 3048, adjuvant therapy initiated within 28-59 days), and late (n = 2136, adjuvant therapy initiated after >59 days) interval cohorts. Median (interquartile range) overall follow-up was 38.6 (24.6-62.0) months. Compared with the reference interval cohort on multivariable analysis, both the early cohort (hazard ratio, 1.17; 95% CI, 1.02-1.35; *P* = .03) and the late cohort (hazard ratio, 1.09; 95% CI, 1.02-1.17; *P* = .008) were associated with worse mortality. Similarly, the reference interval cohort had improved OS compared with the early cohort in 268 propensity-matched pairs (2-year OS, 52.5% [95% CI, 46.7%-59.0%] vs 45.1% [95% CI, 39.5%-51.6%]; *P* = .02) and compared with the late cohort in 2042 propensity-matched pairs (2-year OS, 51.3% [95% CI, 49.1%-53.6%] vs 45.4% [95% CI, 43.3%-47.7%]; *P* = .01). Patients who received adjuvant therapy more than 12 weeks after surgery (n = 683) had improved OS compared with surgery alone in both multivariable analysis (hazard ratio, 0.75; 95% CI, 0.66-0.85; *P* < .001) and 655 propensity-matched pairs (2-year OS, 47.2% [95% CI, 43.5%-51.3%] vs 38.0% [95% CI, 34.4%-42.0%]; *P* < .001).

**Conclusions and Relevance:**

Patients with stage I to II pancreatic cancer who commenced adjuvant therapy within 28 to 59 days after primary surgical resection had improved survival outcomes compared with those with adjuvant therapy before 28 days or after 59 days. Patients who recovered slowly from surgery still benefited from delayed adjuvant therapy initiated more than 12 weeks after surgery compared with patients who underwent surgery only.

## Key Points

**Question:**

Is the timing of adjuvant therapy in resected pancreatic cancer associated with better survival?

**Findings:**

This cohort study of 7548 patients with stage I to II pancreatic cancer in the National Cancer Database suggested improved survival when adjuvant therapy was initiated 28 to 59 days after surgery. Patients who recovered slowly from surgery still benefited from delayed adjuvant therapy initiated more than 12 weeks after the procedure compared with patients who received surgery alone.

**Meaning:**

Shared decision-making between clinicians and patients is needed to individualize when to initiate adjuvant therapy based on patients’ postoperative recovery.

## Teaser

This cohort study evaluates the ideal timing of adjuvant therapy for patients with stage I to II resected pancreatic cancer.

## Introduction

Pancreatic cancer is among the deadliest malignant neoplasms in the United States. It is the fourth leading cause of cancer-related deaths, with 56 770 new cases estimated for 2019.^1^ For early-stage pancreatic cancer, surgery offers patients the best chance of cure, but outcomes remain poor. Despite primary surgery with curative intent, 73% of patients may develop local failure.^2^ Multiple clinical trials previously investigated the clinical outcomes of various adjuvant therapy regimens.^3,4,5,6,7,8,9,10^ A 2008 study sponsored by the Radiation Therapy Oncology Group (RTOG)^6^ suggested the combination of chemotherapy and chemoradiation for select patients, while trials in 2017^7^ and 2018^9^ established the current adjuvant chemotherapy regimens as the standard of care. A current trial, RTOG 0848 (ClinicalTrials.gov identifier: NCT01013649) is examining the role of sequential chemoradiation following adjuvant chemotherapy.

Delays in the initiation of adjuvant treatment or total duration of treatment time have been associated with worse survival outcomes, seen in a wide range of cancers including colorectal,^11^ head and neck,^12^ cervical,^13^ and breast^14^ cancer. While the benefit of adjuvant therapy to patients with resected pancreatic cancer is accepted, its optimal timing after surgery remains under investigation. Prior studies suggested no benefit to early initiation of adjuvant therapy but only compared survival between arbitrarily assigned time periods.^15,16,17,18,19^

This National Cancer Database (NCDB) study compares the outcomes of patients who received adjuvant chemotherapy or chemoradiation at various time intervals, which were defined by a Cox model with restricted cubic splines (RCS).

## Methods

### Patient Population

Patients with pancreatic adenocarcinoma diagnosed from 2004 to 2015 were identified using the NCDB registry. The NCDB is a national cancer database that collects information on approximately 70% of new cancer diagnoses in the United States.^20^ The NCDB provides access to deidentified data sets from Commission on Cancer–accredited programs through online application. Informed consent for this study has been waived because NCDB data are deidentified. The Roswell Park Comprehensive Cancer Center institutional review board exempted our study from institutional review board review. This report follows the Strengthening the Reporting of Observational Studies in Epidemiology (STROBE) reporting guideline.

**Figure 1.**
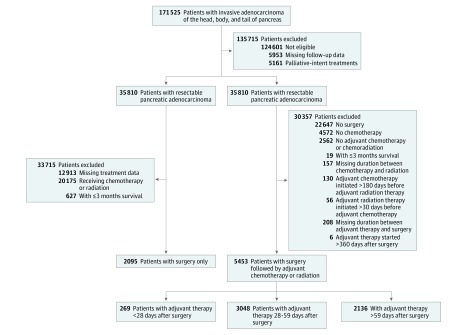
Flow Diagram for Patient Selection Criteria

Our patient selection criteria are detailed in Figure 1. Our initial query resulted in patients with stage I to II, clinical T1-3N0-1M0 pancreatic adenocarcinoma who had been treated with curative-intent resection alone or resection followed by adjuvant chemotherapy or chemoradiation. American Joint Committee on Cancer sixth and seventh editions were used to identify stage I to II disease from 2004 to 2015.

Whipple and Whipple-variant surgical procedures were previously described.^21^ Adjuvant therapy was defined as adjuvant chemotherapy alone or chemotherapy with radiation. Those with adjuvant chemotherapy more than 180 days before adjuvant radiation therapy or with adjuvant radiation therapy more than 30 days before adjuvant chemotherapy were excluded.^22^ Patients with adjuvant therapy more than 360 days after resection were also excluded. Surgical margin was divided into either negative (ie, R0) or positive (ie, R1, R2, or positive margin not otherwise specified). Patients were stratified by age (ie, <67 years or ≥67 years), tumor size (ie, <3.1 cm or ≥3.1 cm), time from diagnosis to surgery (ie, <17 days or ≥17 days), and duration of postoperative inpatient admission (ie, <1 week or ≥1 week) based on median values. The cutoff value of 98 U/mL for cancer antigen (CA) 19-9 levels was predetermined in the NCDB.

All missing values were coded as unknown. To address the immortal time bias, patients with postdiagnosis survival of less than 3 months were excluded as a conditional landmark.^23^ Pertinent prognostic variables, such as performance status, type and duration of chemotherapy, toxicity, and tumor recurrence outcomes, are unavailable in the NCDB. The primary end point was overall survival (OS), defined as time from diagnosis to the last follow-up or death.

### Statistical Analysis

To model the association of time to adjuvant therapy with survival, a multivariable Cox model with RCS was used, as previously shown in other disease sites.^24,25,26^ Restricted cubic splines is a smooth, piecewise polynomial function that evaluates the association of a variable with an outcome without assuming any association a priori.^27,28^ The Cox model with RCS was constructed using 4 knots at the 5th, 35th, 65th, and 95th percentiles, and the 4 knots were determined based on the lowest Akaike information criterion.^27,29^ The model was adjusted for facility type, age, sex, race/ethnicity, insurance, income, residential setting, Charlson/Deyo comorbidity score (CDS), year of diagnosis, primary tumor location within pancreas, tumor grade, tumor size, pathologic T and N stages, CA 19-9 level, surgery type, surgical margin, chemotherapy, radiation therapy, unplanned readmission within 30 days after surgery, duration of inpatient stay after surgery, and time from diagnosis to surgery. The model-derived thresholds were determined based on those at which the hazard ratio (HR) was the smallest with the 95% CIs below an HR of 1.00. These thresholds were used to divide the adjuvant therapy cohort into early, reference, and late interval groups, with the reference interval group having the lowest mortality.

Kaplan-Meier method and log-rank tests were used for OS analysis. Fisher exact test and Mann-Whitney *U* test were used to compare categorical and continuous variables, respectively. Logistic regression univariable analysis and multivariable analysis (MVA) were used to identify potential variables that predicted the delayed use of adjuvant therapy. Cox proportional hazard univariable analysis and MVA were used to identify variables that predicted survival. The MVA was initially built based on all statistically significant variables from the univariable analysis and was finalized using a backward stepwise elimination.

Possible interactions between the treatment and other variables were evaluated using Cox MVA by adding interaction terms.^30^ When the interaction terms were statistically significant, the final Cox MVA model was reanalyzed for each subgroup of variables, and a corresponding forest plot was performed.^30^

To address selection bias, propensity score matching was performed. The reference interval cohort was matched with early and late cohorts separately to examine the association of the timing of adjuvant therapy with survival outcomes. Matched pairs were established by matching baseline characteristics, including facility type, age, CDS, year of diagnosis, tumor grade, tumor size, pathologic T and N stages, CA 19-9 level, surgery type, surgical margin, chemotherapy, radiation therapy dose, time from diagnosis to surgery, duration of postoperative inpatient admission, unplanned readmission within 30 days after surgery, and any additional variable that was statistically significant in Cox MVA. Matching was performed based on nearest neighbor method in a 1:1 ratio without any replacement, with a caliper distance of 0.1 of the standard deviation of the logit of the propensity score.^31^ The standardized difference of each variable was less than 0.1, suggesting the adequate match.^32^

To examine the survival outcome of adjuvant therapy initiated more than 12 weeks after surgery compared with surgery alone, the Cox MVA, Kaplan-Meier method, and propensity score matching were repeated among such cohorts. All analyses were performed using R software version 3.5.1 (R Project for Statistical Computing). All *P* values were 2-sided, and *P* values less than .05 were considered statistically significant.

## Results

### Patient Characteristics

A total of 7548 patients (3770 men [49.9%]; median [interquartile range (IQR)] age, 67 [59-74] years) with resected clinical stage I to II, T1-3N0-1M0 pancreatic adenocarcinoma met inclusion criteria. Of those, 5453 patients (72.2%) were treated with adjuvant therapy, and 2095 (27.8%) were not (Figure 1). Among patients who received adjuvant therapy, 2329 of 5453 patients (42.7%) received it at 40 to 60 days after surgery (eFigure 1 in the Supplement). Median (IQR) follow-up was 38.6 (24.4-62.0) months (eTable 1 in the Supplement).

### Adjuvant Therapy Timing

**Figure 2.**
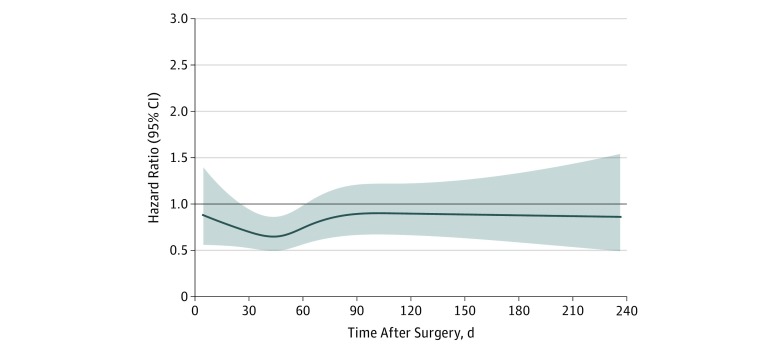
Restricted Cubic Spline Model to Determine Adjuvant Timing With Lowest Mortality Risk The model was adjusted for facility type, age, sex, race/ethnicity, insurance, income, residential setting, Charlson/Deyo comorbidity score, year of diagnosis, primary tumor location within pancreas, tumor grade, tumor size, pathologic T and N stages, cancer antigen 19-9 level, surgery type, surgical margin, chemotherapy, radiation therapy, unplanned readmission within 30 days after surgery, duration of inpatient stay after surgery, and time from diagnosis to surgery. Shaded area indicates 95% CI.

The Cox model with RCS showed the interval with the lowest mortality to be from 28 to 59 days after surgery (Figure 2). Based on these model-determined thresholds, the adjuvant therapy cohort was stratified into 3 intervals: early (n = 269, <28 days), reference (n = 3048, 28-59 days), and late (n = 2136, >59 days) interval cohorts. Early and late cohorts received adjuvant therapy before and after the reference interval, respectively.

On Cox MVA for adjuvant therapy cohorts (eTable 2 in the Supplement), early and late adjuvant therapy cohorts were associated with worse mortality (early: HR, 1.17; 95% CI, 1.02-1.35; *P* = .03; late: HR, 1.09; 95% CI, 1.02-1.17; *P* = .008). After Cox MVA, treatment interactions favored the reference interval for OS compared with the early cohort in patients with worse comorbidities and with unplanned readmission within 30 days after surgery (eFigure 2 in the Supplement). Compared with the late cohort, the reference interval was favored for OS in patients with smaller tumors (eFigure 3 in the Supplement). No treatment interactions were observed in other variables, including age, CDS, pathologic T stage, pathologic N stage, surgical margin, postoperative inpatient admission, unplanned readmission within 30 days after surgery, and time from diagnosis to surgery.

On logistic MVA for initiating adjuvant therapies more than 59 days postoperatively, patients with primary tumor at pancreatic body and tail, multiagent chemotherapy, and radiation therapy were less likely to receive delayed adjuvant therapy (eTable 3 in the Supplement). Patients with older age, black race, lower income, postoperative inpatient admission longer than a week, and unplanned readmission within 30 days after surgery were more likely to have delayed initiation of adjuvant therapy.

**Figure 3.**
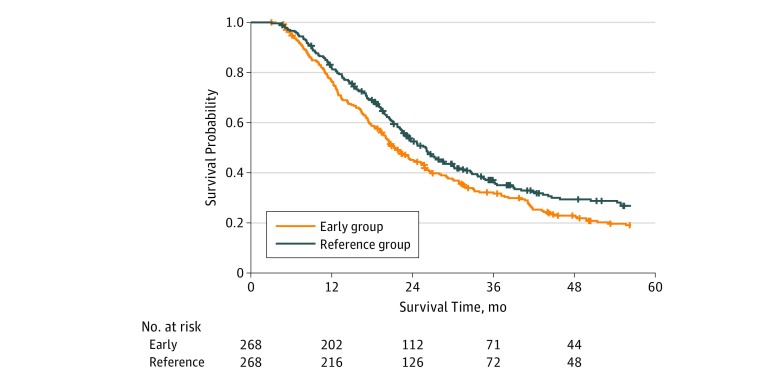
Kaplan-Meier Survival Curves for Early vs Reference Interval After Matching

**Figure 4.**
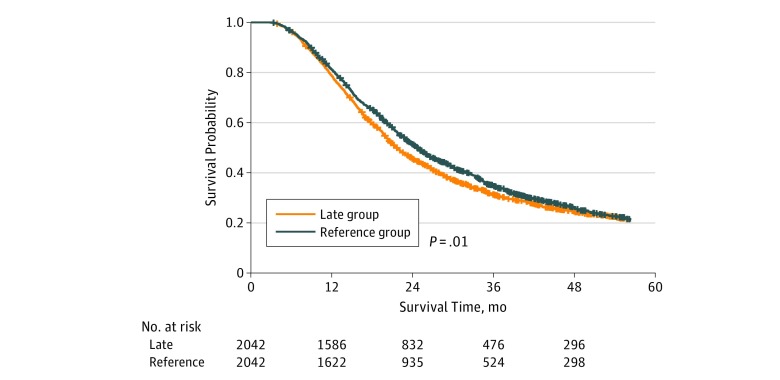
Kaplan-Meier Survival Curves for Late vs Reference Interval After Matching

A total of 268 and 2042 propensity-matched pairs were constructed using early and late cohorts in comparison with the reference interval cohort. All variables were well balanced among these cohorts (eTable 4 in the Supplement). The overall median (IQR) follow-up was 45.6 (25.7-71.6) months for the early cohort and 39.3 (24.9-69.2) months for the reference cohort. The median (IQR) OS was 20.6 (12.2-37.8) months for the early cohort and 23.1 (14.8-38.3) months for the reference cohort (Figure 3). Overall survival at 2 years was 45.1% (95% CI, 39.5%-51.6%) and 52.5% (95% CI, 46.7%-59.0%) for the early and reference cohorts, respectively (*P* = .02). The overall median (IQR) follow-up was 38.9 (24.8-58.8) months for the late cohort and 36.9 (24.1-58.3) months for the reference cohort. The median (IQR) OS was 20.4 (12.8-34.7) months for the late cohort and 22.4 (13.6-36.3) months for the reference cohort (Figure 4). Overall survival at 2 years was 45.4% (95% CI, 43.3%-47.7%) and 51.3% (95% CI, 49.1%-53.6%) for the late and reference cohorts, respectively (*P* = .01).

### Adjuvant Therapy More Than 12 Weeks After Surgery vs Surgery Alone

On Cox MVA (data available on request), adjuvant therapy more than 12 weeks after surgery (HR, 0.75; 95% CI, 0.66-0.85; *P* < .001) was associated with improved survival compared with surgery alone. After Cox MVA, treatment interactions were observed in a node-positive disease subgroup, favoring the adjuvant therapy for OS compared with surgery alone (data available on request). No treatment interactions were seen in other variables, including age, CDS, tumor size, pathologic T stages, surgical margin, duration of postoperative inpatient admission, unplanned readmission within 30 days after surgery, and time from diagnosis to surgery.

A total of 655 propensity-matched pairs were constructed for adjuvant therapy initiated more than 12 weeks after surgery vs surgery alone. All variables were well balanced between these cohorts (data available on request). The overall median (IQR) follow-up was 41.9 (26.2-64.5) months for the adjuvant therapy cohort and 33.6 (21.5-56.3) months for the surgery alone cohort. The median OS was 21.1 (13.1-35.9) months for the adjuvant therapy cohort and 15.5 (7.4-29.3) months for the surgery alone cohort. Overall survival at 2 years was 47.2% (95% CI, 43.5%-51.3%) and 38.0% (95% CI, 34.4%-42.0%) for the adjuvant therapy and surgery alone cohorts, respectively (*P* < .001).

## Discussion

To our knowledge, this is the first study using a multi-institutional national registry to evaluate the timing of adjuvant therapy in resected pancreatic adenocarcinoma across specific time intervals with the reference interval defined by a Cox model with RCS. The results of our study differ from previous reports that found no survival difference when adjuvant therapy was delayed.^15,16,17,18,19^

By examining survival of patients initiating adjuvant therapy before and after set time periods, such as 8 or 12 weeks, these studies may have missed the optimal window for identifying survival benefits. Another factor that may account for this difference is our study included both adjuvant chemotherapy and chemoradiation, while several prior reports focused on adjuvant chemotherapy alone.^15,16,17,18^ After adjusting for radiation therapy on Cox MVA and propensity-matched analysis in our study, the survival benefit of the reference interval remained statistically significant. It is important to note, however, that the European Study Group for Pancreatic Cancer^10^ demonstrated a worse OS in patients treated with adjuvant radiation. Although interpretation of these results was limited by a lack of quality assurance of radiation plans, the increase in toxic effects and shorter time to recurrence with adjuvant radiation highlight the need for careful patient selection. In addition, since the publication of the RTOG study in 2008, 3 weeks of chemotherapy followed by chemoradiation and subsequent chemotherapy were recommended as an option for adjuvant therapy regimen.^6^ Among patients with adjuvant chemoradiation in our study, the median (IQR) duration from the initiation of chemotherapy to the initiation of radiation was 31 (0-70) days. Because our study includes patients diagnosed starting in 2004, the heterogeneity of chemoradiation regimens may in part reflect the patterns of care in the United States. As a result, a subset of patients in our study may not have received the standard of care in accordance with national guidelines.

In both MVA and propensity-matched pairs, our results suggest that patients who received adjuvant therapy more than 12 weeks after surgery had improved OS compared with patients who had surgery alone, supporting findings reported in previous studies.^15,16,17^ Palacio et al^17^ found this survival benefit to persist with a delay in the initiation of adjuvant chemotherapy for as long as 6 months. It is not clear from our NCDB analysis if delayed adjuvant therapy is palliative intent.

In our study, patients with more comorbidities and unplanned postoperative readmission favored the reference interval compared with the early cohort. Such patients may have had more postoperative complications and likely needed additional time to recover rather than starting adjuvant therapy as early as possible.^33^ Our logistic MVA similarly showed those with older age, prolonged postoperative inpatient admission, and unplanned postoperative readmission were more likely to have delayed adjuvant therapy.

In our interaction term analysis, the reference interval was favored compared with the late cohort among patients with smaller tumor size. In addition, adjuvant therapy given more than 12 weeks after surgery was favored compared with surgery alone among patients with lymph node metastases. A 2018 study^34^ described a subgroup of small pancreatic tumors with lymph node metastases that may have more aggressive cancer biology, which may benefit from either receiving adjuvant therapy promptly or avoiding surgery alone.

In our Cox MVA results, being diagnosed in more recent years was associated with improved survival. This is likely in part owing to recent improvements in adjuvant chemotherapy regimens, such as FOLFIRINOX (folinic acid, fluorouracil, irinotecan, and oxaliplatin) and the combination of gemcitabine and capecitabine, with improved survival as shown in clinical trials.^7,9^ Our study also showed that older age, lower income, rural residential setting, elevated CA 19-9 levels, and positive surgical margin were associated with worse mortality, and being African American was associated with delayed adjuvant therapy. These results are consistent with prior studies that found that socioeconomic status plays a role in treatment outcomes despite similar tumor characteristics.^35,36,37,38,39^

### Limitations

This study has limitations. As a national registry–based study, it is limited by missing patient information, documentation error, and its retrospective nature. Detailed information regarding chemotherapy and chemoradiation regimens are unavailable in the NCDB. Importantly, the reason for early or late initiation of adjuvant therapy was unknown. Those who received adjuvant therapy late may have had postoperative complications or poor performance status, delaying the initiation of treatment. Toxicity profile and performance status are not included in the NCDB. To minimize selection bias, we performed propensity score matching for baseline characteristics, including unplanned postoperative readmissions and duration of postoperative inpatient admission, as proxy measures for postoperative performance status and complications.^40,41,42^ While those with unplanned readmissions and prolonged postoperative inpatient admission were more likely to receive delayed adjuvant therapy based on our logistic MVA, they were not statistically significant for worse mortality in Cox MVA. Despite these limitations, the NCDB provides data on large numbers of patients with pancreatic cancer not otherwise available through single-institutional studies.

## Conclusions

This study used a multi-institutional national registry to evaluate the timing of adjuvant therapy in resected pancreatic adenocarcinoma across specific time intervals. To our knowledge, it is the first study to suggest that patients who commence adjuvant therapy within 28 to 59 days after primary surgical resection of pancreatic adenocarcinoma have improved survival outcomes compared with those who waited for more than 59 days. However, patients who recover slowly from surgery may still benefit from delayed adjuvant therapy initiated more than 12 weeks after surgery. Further studies examining the optimal treatment strategy following surgery in this challenging population are warranted.

## Notes

Supplement. **eFigure 1.** Histogram for Frequency of Patients Based on Days After Surgery **eFigure 2.** Forest Plot for Subgroup Analysis Between Early vs Reference Interval Cohorts **eFigure 3.** Forest Plot for Subgroup Analysis Between Late vs Reference Interval Cohorts **eTable 1.** Baseline Characteristics **eTable 2. **Cox MVA for Adjuvant Therapy Cohorts **eTable 3.** Logistic MVA for Initiating Adjuvant Therapies More Than 59 Days After Surgery **eTable 4.** Baseline Characteristics of Matched Cohorts for Adjuvant Therapy
