# Supplementary material for: Association of Timing of Adjuvant Therapy With Survival in Patients With Resected Stage I to II Pancreatic Cancer
Source: JAMA Netw Open. 2019 Aug 14;2(8):e199126. doi: 10.1001/jamanetworkopen.2019.9126 (PMC6694394; doi:10.1001/jamanetworkopen.2019.9126)
Supplement: Supplement. — eFigure 1. Histogram for Frequency of Patients Based on Days After Surgery eFigure 2. Forest Plot for Subgroup Analysis Between Early vs Reference Interval Cohorts eFigure 3. Forest Plot for Subgroup Analysis Between Late vs Reference Interval Cohorts eTable 1. Baseline Characteristics eTable 2. Cox MVA for Adjuvant Therapy Cohorts eTable 3. Logistic MVA for Initiating Adjuvant Therapies More Than 59 Days After Surgery eTable 4. Baseline Characteristics of Matched Cohorts for Adjuvant Therapy [file jamanetwopen-2-e199126-s001.pdf]

# Supplementary Online Content

Ma SJ, Oladeru OT, Miccio JA, Iovoli AJ, Hermann GM, Singh AK. Association of timing of adjuvant therapy with survival in patients with resected stage I to II pancreatic cancer. *JAMA Netw Open*. 2019;2(8):e199126. doi:10.1001/jamanetworkopen.2019.9126

**eFigure 1.** Histogram for Frequency of Patients Based on Days After Surgery
**eFigure 2.** Forest Plot for Subgroup Analysis Between Early vs Reference Interval Cohorts
**eFigure 3.** Forest Plot for Subgroup Analysis Between Late vs Reference Interval Cohorts
**eTable 1.** Baseline Characteristics
**eTable 2.** Cox MVA for Adjuvant Therapy Cohorts
**eTable 3.** Logistic MVA for Initiating Adjuvant Therapies More Than 59 Days After Surgery
**eTable 4.** Baseline Characteristics of Matched Cohorts for Adjuvant Therapy

This supplementary material has been provided by the authors to give readers additional information about their work.

© 2019 Ma SJ et al. *JAMA Network Open.*

**eFigure 1.** Histogram for Frequency of Patients Based on Days After Surgery

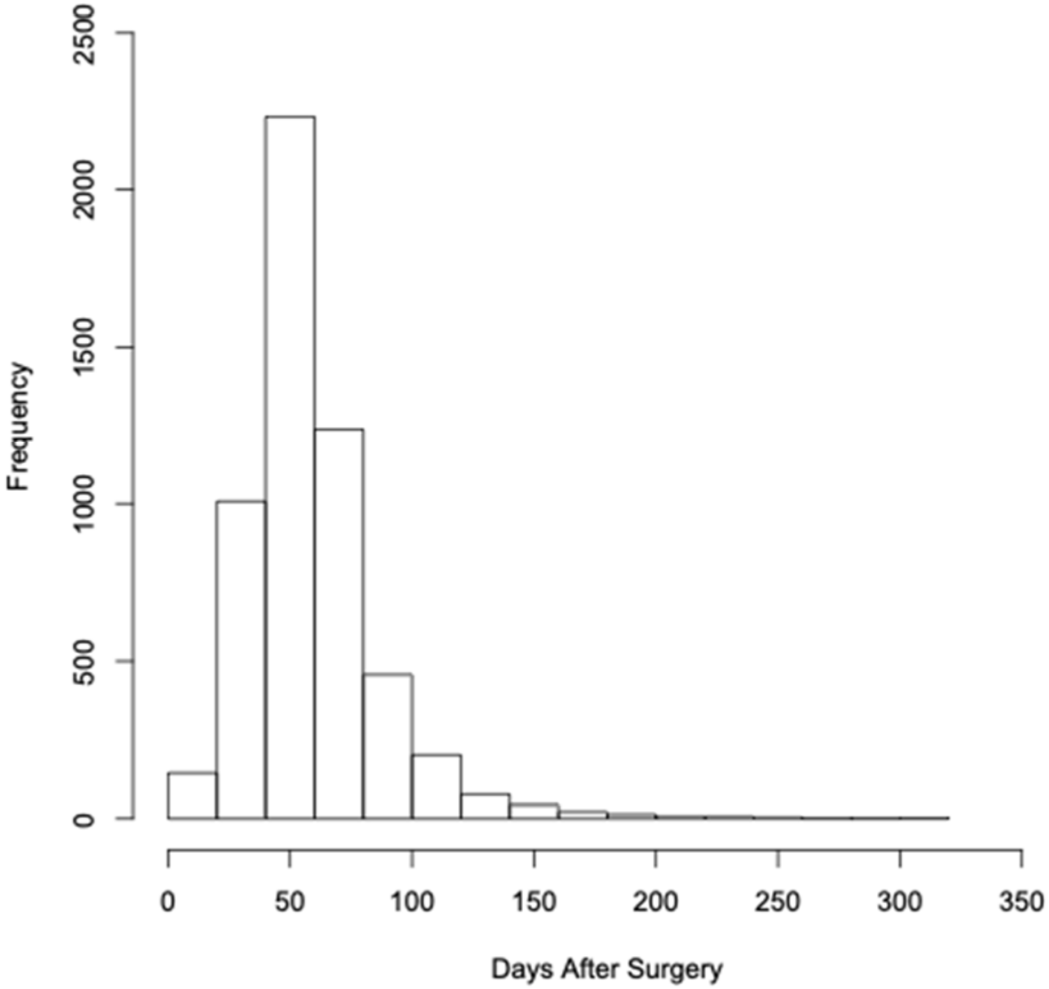


© 2019 Ma SJ et al. *JAMA Network Open.*

**eFigure 2.** Forest Plot for Subgroup Analysis Between Early vs Reference Interval Cohorts

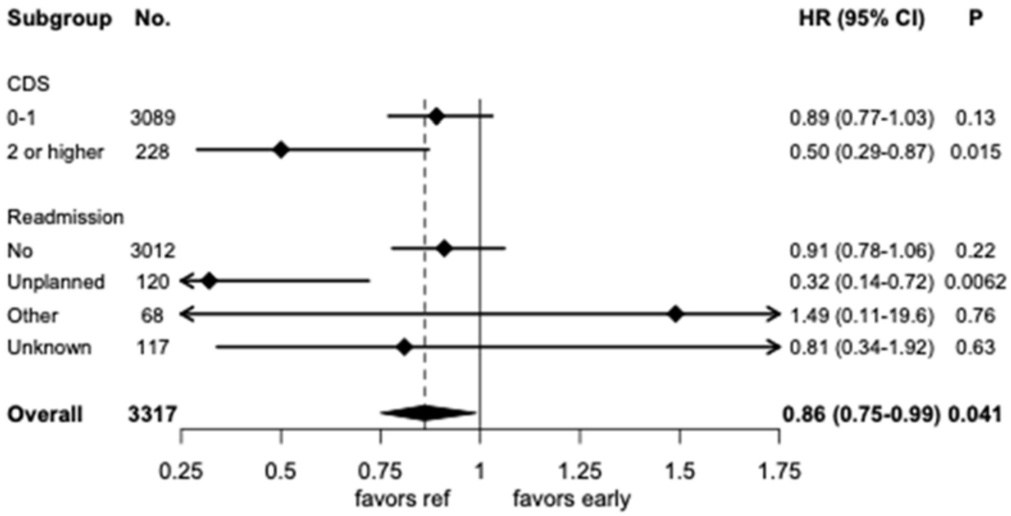


Ref: reference interval; early: early interval; CDS: Charlson-Deyo comorbidity score

© 2019 Ma SJ et al. *JAMA Network Open.*

**eFigure 3.** Forest Plot for Subgroup Analysis Between Late vs Reference Interval Cohorts

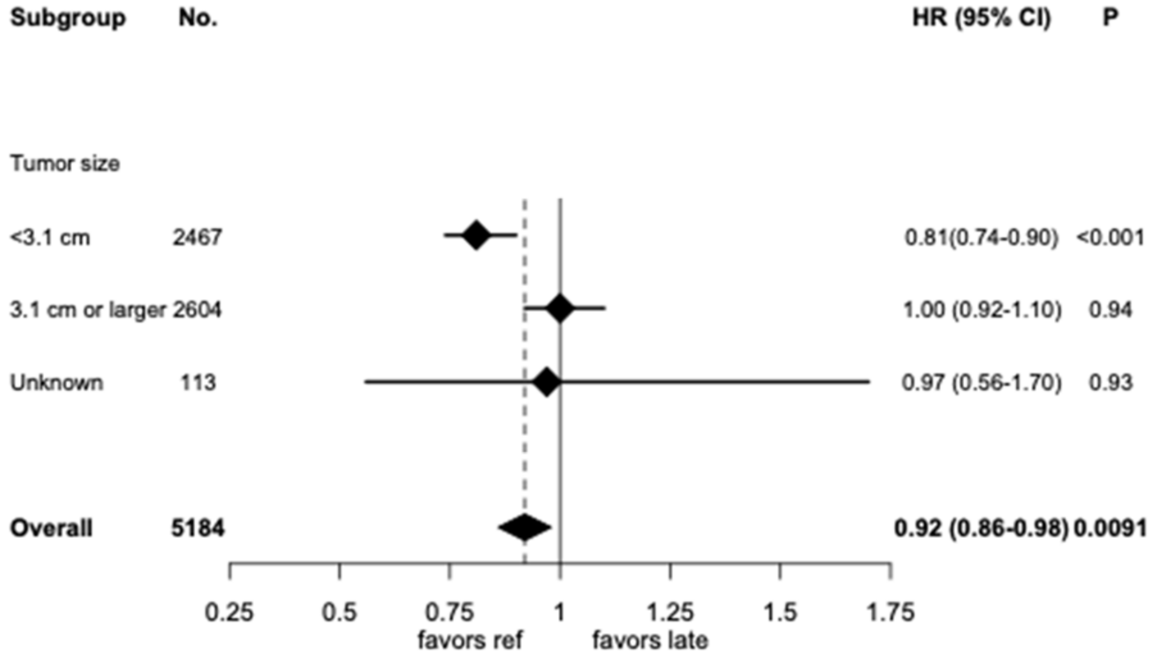


Ref: reference interval; late: late interval

© 2019 Ma SJ et al. *JAMA Network Open.*

## **eTable 1.** Baseline Characteristics

| | Adjuvant Therapy | | | | | | | Surgery only | | Adjuvant Therapy | | |
|---|---|---|---|---|---|---|---|---|---|---|---|---|
| | Ref | | Early | | Late | | | | | >12 weeks | | |
| | N | % | N | % | N | % | P | N | % | N | % | P |
| Facility | | | | | | | 0.02 | | | | | <0.001 |
| Nonacademic | 1710 | 56 | 169 | 63 | 1149 | 54 | | 993 | 47 | 379 | 55 | |
| Academic | 1311 | 43 | 100 | 37 | 971 | 45 | | 1090 | 52 | 299 | 44 | |
| NA | 27 | 1 | 0 | 0 | 16 | 1 | | 12 | 1 | 5 | 1 | |
| | | | | | | | | | | | | |
| Age | | | | | | | <0.001 | | | | | <0.001 |
| <67 | 1687 | 55 | 166 | 62 | 1018 | 48 | | 775 | 37 | 319 | 47 | |
| ≥67 | 1361 | 45 | 103 | 38 | 1118 | 52 | | 1320 | 63 | 364 | 53 | |
| NA | 0 | 0 | 0 | 0 | 0 | 0 | | 0 | 0 | 0 | 0 | |
| | | | | | | | | | | | | |
| Gender | | | | | | | 0.04 | | | | | 0.57 |
| Female | 1465 | 48 | 144 | 54 | 1090 | 51 | | 1079 | 52 | 343 | 50 | |
| Male | 1583 | 52 | 125 | 46 | 1046 | 49 | | 1016 | 48 | 340 | 50 | |
| NA | 0 | 0 | 0 | 0 | 0 | 0 | | 0 | 0 | 0 | 0 | |
| | | | | | | | | | | | | |
| Race | | | | | | | <0.001 | | | | | 0.64 |
| White | 2699 | 89 | 235 | 87 | 1812 | 85 | | 1808 | 86 | 590 | 86 | |
| Black | 239 | 8 | 26 | 10 | 237 | 11 | | 194 | 9 | 71 | 10 | |
| Other | 81 | 3 | 6 | 2 | 71 | 3 | | 64 | 3 | 18 | 3 | |
| NA | 29 | 1 | 2 | 1 | 16 | 1 | | 29 | 1 | 4 | 1 | |
| | | | | | | | | | | | | |
| Insurance | | | | | | | <0.001 | | | | | 0.04 |
| None | 70 | 2 | 6 | 2 | 56 | 3 | | 49 | 2 | 15 | 2 | |
| Nonprivate | 1671 | 55 | 132 | 49 | 1318 | 62 | | 1414 | 67 | 430 | 63 | |
| Private | 1286 | 42 | 127 | 47 | 745 | 35 | | 606 | 29 | 234 | 34 | |
| NA | 21 | 1 | 4 | 1 | 17 | 1 | | 26 | 1 | 4 | 1 | |
| | | | | | | | | | | | | |
| Income | | | | | | | 0.01 | | | | | 0.28 |
| Above median | 1946 | 64 | 168 | 62 | 1281 | 60 | | 1126 | 54 | 391 | 57 | |
| Below median | 1060 | 35 | 93 | 35 | 830 | 39 | | 910 | 43 | 286 | 42 | |
| NA | 42 | 1 | 8 | 3 | 25 | 1 | | 59 | 3 | 6 | 1 | |
| | | | | | | | | | | | | |
| Residential setting | | | | | | | 0.51 | | | | | 0.24 |
| Metro | 2438 | 80 | 218 | 81 | 1738 | 81 | | 1591 | 76 | 550 | 81 | |
| Urban | 447 | 15 | 31 | 12 | 292 | 14 | | 347 | 17 | 98 | 14 | |
| Rural | 50 | 2 | 4 | 1 | 43 | 2 | | 43 | 2 | 16 | 2 | |
| NA | 113 | 4 | 16 | 6 | 63 | 3 | | 114 | 5 | 19 | 3 | |
| | | | | | | | | | | | | |
| Charlson-Deyo Score | | | | | | | 0.83 | | | | | 0.51 |
| 0-1 | 2840 | 93 | 249 | 93 | 1995 | 93 | | 1927 | 92 | 634 | 93 | |
| ≥2 | 208 | 7 | 20 | 7 | 141 | 7 | | 168 | 8 | 49 | 7 | |
| NA | 0 | 0 | 0 | 0 | 0 | 0 | | 0 | 0 | 0 | 0 | |

© 2019 Ma SJ et al. *JAMA Network Open.*

| | | | | | | | | | | | | |
|---|---|---|---|---|---|---|---|---|---|---|---|---|
| | | | | | | | | | | | | |
| Year of diagnosis | | | | | | | <0.001 | | | | | <0.001 |
| 2004-2007 | 228 | 7 | 30 | 11 | 182 | 9 | | 539 | 26 | 59 | 9 | |
| 2008-2011 | 1487 | 49 | 155 | 58 | 997 | 47 | | 981 | 47 | 316 | 46 | |
| 2012-2015 | 1333 | 44 | 84 | 31 | 957 | 45 | | 575 | 27 | 308 | 45 | |
| NA | 0 | 0 | 0 | 0 | 0 | 0 | | 0 | 0 | 0 | 0 | |
| | | | | | | | | | | | | |
| Primary tumor site | | | | | | | <0.001 | | | | | 0.20 |
| Head | 2418 | 79 | 190 | 71 | 1793 | 84 | | 1652 | 79 | 552 | 81 | |
| Body | 237 | 8 | 29 | 11 | 134 | 6 | | 180 | 9 | 44 | 6 | |
| Tail | 393 | 13 | 50 | 19 | 209 | 10 | | 263 | 13 | 87 | 13 | |
| NA | 0 | 0 | 0 | 0 | 0 | 0 | | 0 | 0 | 0 | 0 | |
| | | | | | | | | | | | | |
| Tumor grade | | | | | | | 0.18 | | | | | 0.51 |
| Well diff | 232 | 8 | 19 | 7 | 159 | 7 | | 171 | 8 | 53 | 8 | |
| Mod diff | 1476 | 48 | 121 | 45 | 1036 | 49 | | 991 | 47 | 348 | 51 | |
| Poor diff | 1091 | 36 | 104 | 39 | 761 | 36 | | 688 | 33 | 231 | 34 | |
| Other | 42 | 1 | 0 | 0 | 16 | 1 | | 28 | 1 | 5 | 1 | |
| NA | 207 | 7 | 25 | 9 | 164 | 8 | | 217 | 10 | 46 | 7 | |
| | | | | | | | | | | | | |
| Tumor size (cm) | | | | | | | 0.05 | | | | | 0.53 |
| <3.1 | 1411 | 46 | 120 | 45 | 1056 | 49 | | 1059 | 51 | 358 | 52 | |
| ≥3.1 | 1572 | 52 | 141 | 52 | 1032 | 48 | | 968 | 46 | 309 | 45 | |
| NA | 65 | 2 | 8 | 3 | 48 | 2 | | 68 | 3 | 16 | 2 | |
| | | | | | | | | | | | | |
| Pathologic T stage | | | | | | | 0.01 | | | | | <0.001 |
| 1 | 111 | 4 | 10 | 4 | 112 | 5 | | 184 | 9 | 37 | 5 | |
| 2 | 403 | 13 | 47 | 17 | 256 | 12 | | 349 | 17 | 86 | 13 | |
| 3 | 2359 | 77 | 186 | 69 | 1636 | 77 | | 1336 | 64 | 519 | 76 | |
| 4 | 41 | 1 | 6 | 2 | 30 | 1 | | 35 | 2 | 9 | 1 | |
| Other | 1 | 0 | 0 | 0 | 0 | 0 | | 3 | 0 | 0 | 0 | |
| NA | 133 | 4 | 20 | 7 | 102 | 5 | | 188 | 9 | 32 | 5 | |
| | | | | | | | | | | | | |
| Pathologic N stage | | | | | | | 0.27 | | | | | <0.001 |
| 0 | 817 | 27 | 62 | 23 | 602 | 28 | | 821 | 39 | 217 | 32 | |
| 1 | 2078 | 68 | 182 | 68 | 1423 | 67 | | 1075 | 51 | 432 | 63 | |
| NA | 153 | 5 | 25 | 9 | 111 | 5 | | 199 | 9 | 34 | 5 | |
| | | | | | | | | | | | | |
| CA 19-9 | | | | | | | 0.05 | | | | | 0.63 |
| <98 U/mL | 799 | 26 | 55 | 20 | 514 | 24 | | 310 | 15 | 159 | 23 | |
| ≥98 U/mL | 658 | 22 | 33 | 12 | 491 | 23 | | 285 | 14 | 157 | 23 | |
| NA | 1591 | 52 | 181 | 67 | 1131 | 53 | | 1500 | 72 | 367 | 54 | |
| | | | | | | | | | | | | |
| Surgery | | | | | | | 0.006 | | | | | 0.66 |
| Whipple variant | 904 | 30 | 75 | 28 | 629 | 29 | | 590 | 28 | 204 | 30 | |
| Whipple | 1455 | 48 | 112 | 42 | 1067 | 50 | | 1007 | 48 | 317 | 46 | |
| Other | 689 | 23 | 82 | 30 | 440 | 21 | | 498 | 24 | 162 | 24 | |

© 2019 Ma SJ et al. *JAMA Network Open.*

| | | | | | | | | | | | | |
|---|---|---|---|---|---|---|---|---|---|---|---|---|
| NA | 0 | 0 | 0 | 0 | 0 | 0 | | 0 | 0 | 0 | 0 | |
| | | | | | | | | | | | | |
| Surgical margin | | | | | | | 0.29 | | | | | <0.001 |
| Negative | 2318 | 76 | 190 | 71 | 1618 | 76 | | 1664 | 79 | 508 | 74 | |
| Positive | 659 | 22 | 68 | 25 | 469 | 22 | | 354 | 17 | 160 | 23 | |
| NA | 71 | 2 | 11 | 4 | 49 | 2 | | 77 | 4 | 15 | 2 | |
| | | | | | | | | | | | | |
| Chemotherapy | | | | | | | <0.001 | | | | | <0.001 |
| None | 0 | 0 | 0 | 0 | 0 | 0 | | 2095 | 100 | 0 | 0 | |
| Single agent | 2082 | 68 | 158 | 59 | 1578 | 74 | | 0 | 0 | 521 | 76 | |
| Multi agent | 966 | 32 | 111 | 41 | 558 | 26 | | 0 | 0 | 162 | 24 | |
| NA | 0 | 0 | 0 | 0 | 0 | 0 | | 0 | 0 | 0 | 0 | |
| | | | | | | | | | | | | |
| Radiation | | | | | | | <0.001 | | | | | <0.001 |
| No | 1511 | 50 | 124 | 46 | 1198 | 56 | | 2095 | 100 | 425 | 62 | |
| Yes | 1537 | 50 | 145 | 54 | 938 | 44 | | 0 | 0 | 258 | 38 | |
| NA | 0 | 0 | 0 | 0 | 0 | 0 | | 0 | 0 | 0 | 0 | |
| | | | | | | | | | | | | |
| Radiation dose (Gy) | | | | | | | 0.65, 0.60[a] | | | | | NA |
| Median | 50.4 | | 50.4 | | 50.4 | | | NA | | 50.4 | | |
| IQR | 50.0-50.4 | | 46.8-54.0 | | 49.6-50.4 | | | NA | | 50.0-50.4 | | |
| | | | | | | | | | | | | |
| Inpatient stay postoperatively | | | | | | | <0.001 | | | | | 0.05 |
| <1 week | 905 | 30 | 103 | 38 | 464 | 22 | | 416 | 20 | 147 | 22 | |
| ≥1 week | 1717 | 56 | 130 | 48 | 1323 | 62 | | 1494 | 71 | 424 | 62 | |
| NA | 426 | 14 | 36 | 13 | 349 | 16 | | 185 | 9 | 112 | 16 | |
| | | | | | | | | | | | | |
| Time from dx to surgery | | | | | | | 0.37 | | | | | 0.10 |
| <17 days | 1529 | 50 | 147 | 55 | 1084 | 51 | | 995 | 47 | 355 | 52 | |
| ≥17 days | 1519 | 50 | 122 | 45 | 1052 | 49 | | 1063 | 51 | 328 | 48 | |
| NA | 0 | 0 | 0 | 0 | 0 | 0 | | 37 | 2 | 0 | 0 | |
| | | | | | | | | | | | | |
| Unplanned readmission within 30 days | | | | | | | <0.001 | | | | | 0.14 |
| No | 2767 | 91 | 245 | 91 | 1848 | 87 | | 1837 | 88 | 572 | 84 | |
| Yes | 110 | 4 | 10 | 4 | 179 | 8 | | 195 | 9 | 80 | 12 | |
| Others | 64 | 2 | 4 | 1 | 30 | 1 | | 31 | 1 | 10 | 1 | |
| NA | 107 | 4 | 10 | 4 | 79 | 4 | | 32 | 2 | 21 | 3 | |

[a]Total radiation dose P value: reference vs early interval cohorts, reference vs late interval cohorts
N: number or count; NA: not available or unknown; IQR: interquartile range; diff: differentiated

© 2019 Ma SJ et al. *JAMA Network Open.*

**eTable 2.** Cox MVA for Adjuvant Therapy Cohorts

| | HR | 95% CI | P |
|---|---|---|---|
| Facility | | | |
| Nonacademic | 1 | Ref | |
| Academic | 0.85 | 0.80-0.91 | <0.001 |
| | | | |
| Age | | | |
| <67 | 1 | Ref | |
| ≥67 | 1.15 | 1.08-1.23 | <0.001 |
| | | | |
| Income | | | |
| Above median | 1 | Ref | |
| Below median | 1.11 | 1.04-1.19 | 0.003 |
| | | | |
| Residential setting | | | |
| Metro | 1 | Ref | |
| Urban | 1.03 | 0.93-1.13 | 0.59 |
| Rural | 1.26 | 1.01-1.58 | 0.04 |
| | | | |
| Charlson-Deyo Score | | | |
| 0-1 | 1 | Ref | |
| ≥2 | 1.20 | 1.07-1.36 | 0.003 |
| | | | |
| Year of diagnosis | | | |
| 2004-2007 | 1 | Ref | |
| 2008-2011 | 0.93 | 0.83-1.04 | 0.20 |
| 2012-2015 | 0.84 | 0.74-0.95 | 0.007 |
| | | | |
| Tumor grade | | | |
| Well diff | 1 | Ref | |
| Mod diff | 1.16 | 1.02-1.32 | 0.02 |
| Poor diff | 1.45 | 1.27-1.65 | <0.001 |
| Other | 1.16 | 0.82-1.65 | 0.41 |
| | | | |
| Tumor size (cm) | | | |
| <3.1 | 1 | Ref | |
| ≥3.1 | 1.29 | 1.21-1.38 | <0.001 |
| | | | |
| Pathologic T stage | | | |
| 1-2 | 0.86 | 0.79-0.94 | 0.001 |
| 3-4 | 1 | Ref | |
| | | | |
| Pathologic N stage | | | |
| 0 | 1 | Ref | |
| 1 | 1.54 | 1.42-1.66 | <0.001 |
| | | | |
| CA 19-9 | | | |

© 2019 Ma SJ et al. *JAMA Network Open.*

| | | | |
|---|---|---|---|
| <98 U/mL | 1 | Ref | |
| ≥98 U/mL | 1.28 | 1.16-1.41 | <0.001 |
| | | | |
| Surgical margin | | | |
| Negative | 1 | Ref | |
| Positive | 1.49 | 1.39-1.61 | <0.001 |
| | | | |
| Radiation | | | |
| No | 1 | Ref | |
| Yes | 0.87 | 0.81-0.92 | <0.001 |
| | | | |
| Inpatient stay postoperatively | | | |
| <1 week | 1 | Ref | |
| ≥1 week | 1.10 | 1.02-1.18 | 0.02 |
| | | | |
| Adjuvant therapy timing | | | |
| Ref | 1 | Ref | |
| Early | 1.17 | 1.02-1.35 | 0.03 |
| Late | 1.09 | 1.02-1.17 | 0.008 |

HR: hazard ratio; CI: confidence interval; diff: differentiated; ref: reference; early: early interval cohort; late: late interval cohort

© 2019 Ma SJ et al. *JAMA Network Open.*

**eTable 3.** Logistic MVA for Initiating Adjuvant Therapies More Than 59 Days After Surgery

| | **OR** | **95% CI** | **P** |
|---|---|---|---|
| Age | | | |
| <67 | 1 | Ref | |
| ≥67 | 1.08 | 1.05-1.11 | <0.001 |
| | | | |
| Race | | | |
| White | 1 | Ref | |
| Black | 1.10 | 1.05-1.15 | <0.001 |
| Other | 1.32 | 0.96-1.82 | 0.09 |
| | | | |
| Income | | | |
| Above median | 1 | Ref | |
| Below median | 1.03 | 1.004-1.06 | 0.03 |
| | | | |
| Primary tumor site | | | |
| Head | 1 | Ref | |
| Body | 0.94 | 0.89-0.98 | 0.009 |
| Tail | 0.93 | 0.89-0.97 | <0.001 |
| | | | |
| Chemotherapy | | | |
| Single agent | 1 | Ref | |
| Multi agent | 0.95 | 0.92-0.98 | <0.001 |
| | | | |
| Radiation | | | |
| No | 1 | Ref | |
| Yes | 0.95 | 0.93-0.98 | <0.001 |
| | | | |
| Inpatient stay postoperatively | | | |
| <1 week | 1 | Ref | |
| ≥1 week | 1.08 | 1.05-1.12 | <0.001 |
| | | | |
| Unplanned readmission within 30 days | | | |
| No | 1 | Ref | |
| Yes | 1.24 | 1.18-1.32 | <0.001 |
| Others | 0.72 | 0.46-1.10 | 0.14 |

OR: Odds ratio; CI: confidence interval; ref: reference

© 2019 Ma SJ et al. *JAMA Network Open.*

**eTable 4.** Baseline Characteristics of Matched Cohorts for Adjuvant Therapy

| | Adjuvant Therapy | | | | | | | | | |
|---|---|---|---|---|---|---|---|---|---|---|
| | **Ref** | | **Early** | | | **Ref** | | **Late** | | |
| | **N** | **%** | **N** | **%** | **P** | **N** | **%** | **N** | **%** | **P** |
| Facility | | | | | 0.48 | | | | | 0.44 |
| Nonacademic | 168 | 63 | 159 | 59 | | 1107 | 54 | 1147 | 56 | |
| Academic | 100 | 37 | 109 | 41 | | 920 | 45 | 880 | 43 | |
| NA | 0 | 0 | 0 | 0 | | 15 | 1 | 15 | 1 | |
| | | | | | | | | | | |
| Age | | | | | 0.12 | | | | | 0.66 |
| <67 | 165 | 62 | 146 | 54 | | 997 | 49 | 1012 | 50 | |
| ≥67 | 103 | 38 | 122 | 46 | | 1045 | 51 | 1030 | 50 | |
| NA | 0 | 0 | 0 | 0 | | 0 | 0 | 0 | 0 | |
| | | | | | | | | | | |
| Income | | | | | 0.84 | | | | | 0.19 |
| Above median | 167 | 62 | 161 | 60 | | 1241 | 61 | 1292 | 63 | |
| Below median | 93 | 35 | 100 | 37 | | 777 | 38 | 722 | 35 | |
| NA | 8 | 3 | 7 | 3 | | 24 | 1 | 28 | 1 | |
| | | | | | | | | | | |
| Residential setting | | | | | 0.89 | | | | | 0.76 |
| Metro | 217 | 81 | 222 | 83 | | 1660 | 81 | 1644 | 81 | |
| Urban | 31 | 12 | 28 | 10 | | 283 | 14 | 284 | 14 | |
| Rural | 4 | 1 | 5 | 2 | | 38 | 2 | 42 | 2 | |
| NA | 16 | 6 | 13 | 5 | | 61 | 3 | 72 | 4 | |
| | | | | | | | | | | |
| Charlson-Deyo Score | | | | | 0.87 | | | | | 0.25 |
| 0-1 | 248 | 93 | 246 | 92 | | 1907 | 93 | 1887 | 92 | |
| ≥2 | 20 | 7 | 22 | 8 | | 135 | 7 | 155 | 8 | |
| NA | 0 | 0 | 0 | 0 | | 0 | 0 | 0 | 0 | |
| | | | | | | | | | | |
| Year of diagnosis | | | | | 0.71 | | | | | 0.91 |
| 2004-2007 | 30 | 11 | 31 | 12 | | 172 | 8 | 167 | 8 | |
| 2008-2011 | 154 | 57 | 145 | 54 | | 958 | 47 | 970 | 48 | |
| 2012-2015 | 84 | 31 | 92 | 34 | | 912 | 45 | 905 | 44 | |
| NA | 0 | 0 | 0 | 0 | | 0 | 0 | 0 | 0 | |
| | | | | | | | | | | |
| Tumor grade | | | | | 0.44 | | | | | 0.85 |
| Well diff | 19 | 7 | 14 | 5 | | 155 | 8 | 160 | 8 | |
| Mod diff | 121 | 45 | 139 | 52 | | 989 | 48 | 976 | 48 | |
| Poor diff | 104 | 39 | 92 | 34 | | 732 | 36 | 723 | 35 | |
| Other | 24 | 9 | 23 | 9 | | 16 | 1 | 21 | 1 | |
| NA | 0 | 0 | 0 | 0 | | 150 | 7 | 162 | 8 | |
| | | | | | | | | | | |
| Tumor size (cm) | | | | | 0.07 | | | | | 0.94 |
| <3.1 | 120 | 45 | 123 | 46 | | 998 | 49 | 988 | 48 | |
| ≥3.1 | 140 | 52 | 144 | 54 | | 997 | 49 | 1008 | 49 | |

© 2019 Ma SJ et al. *JAMA Network Open.*

| | | | | | | | | | | |
|---|---|---|---|---|---|---|---|---|---|---|
| NA | 8 | 3 | 1 | 0 | | 47 | 2 | 46 | 2 | |
| | | | | | | | | | | |
| Pathologic T stage | | | | | 0.50 | | | | | 0.94 |
| 1-2 | 57 | 21 | 68 | 25 | | 351 | 17 | 346 | 17 | |
| 3-4 | 192 | 72 | 180 | 67 | | 1595 | 78 | 1596 | 78 | |
| NA | 19 | 7 | 20 | 7 | | 96 | 5 | 100 | 5 | |
| | | | | | | | | | | |
| Pathologic N stage | | | | | 0.89 | | | | | 0.92 |
| 0 | 62 | 23 | 59 | 22 | | 577 | 28 | 578 | 28 | |
| 1 | 182 | 68 | 182 | 68 | | 1360 | 67 | 1353 | 66 | |
| NA | 24 | 9 | 27 | 10 | | 105 | 5 | 111 | 5 | |
| | | | | | | | | | | |
| CA 19-9 | | | | | 0.74 | | | | | 0.38 |
| <98 U/mL | 55 | 21 | 61 | 23 | | 496 | 24 | 521 | 26 | |
| ≥98 U/mL | 33 | 12 | 36 | 13 | | 467 | 23 | 433 | 21 | |
| NA | 180 | 67 | 171 | 64 | | 1079 | 53 | 1088 | 53 | |
| | | | | | | | | | | |
| Surgery | | | | | 0.28 | | | | | 0.93 |
| Whipple variant | 75 | 28 | 59 | 22 | | 605 | 30 | 606 | 30 | |
| Whipple | 112 | 42 | 124 | 46 | | 1007 | 49 | 997 | 49 | |
| Other | 81 | 30 | 85 | 32 | | 430 | 21 | 439 | 21 | |
| | | | | | | | | | | |
| Surgical margin | | | | | 0.86 | | | | | 0.88 |
| Negative | 190 | 71 | 192 | 72 | | 1548 | 76 | 1539 | 75 | |
| Positive | 68 | 25 | 64 | 24 | | 449 | 22 | 461 | 23 | |
| NA | 10 | 4 | 12 | 4 | | 45 | 2 | 42 | 2 | |
| | | | | | | | | | | |
| Chemotherapy | | | | | 0.66 | | | | | 0.20 |
| Single agent | 157 | 59 | 163 | 61 | | 1493 | 73 | 1455 | 71 | |
| Multi agent | 111 | 41 | 105 | 39 | | 549 | 27 | 587 | 29 | |
| | | | | | | | | | | |
| Radiation | | | | | 0.93 | | | | | 0.12 |
| No | 123 | 46 | 125 | 47 | | 1123 | 55 | 1072 | 52 | |
| Yes | 145 | 54 | 143 | 53 | | 919 | 45 | 970 | 48 | |
| | | | | | | | | | | |
| Radiation dose (Gy) | | | | | 0.98 | | | | | 0.06 |
| Median | 50.4 | | 50.4 | | | 50.4 | | 50.4 | | |
| IQR | 46.8-54.0 | | 50.4-54.0 | | | 48.6-50.4 | | 50.0-52.2 | | |
| | | | | | | | | | | |
| Time from dx to surgery | | | | | 0.54 | | | | | 0.14 |
| <17 days | 146 | 54 | 138 | 51 | | 1044 | 51 | 996 | 49 | |
| ≥17 days | 122 | 46 | 130 | 49 | | 998 | 49 | 1046 | 51 | |
| | | | | | | | | | | |
| Inpatient stay postoperatively | | | | | 0.97 | | | | | 0.43 |
| <1 week | 102 | 38 | 104 | 39 | | 460 | 23 | 494 | 24 | |
| ≥1 week | 130 | 49 | 127 | 47 | | 1249 | 61 | 1215 | 60 | |
| NA | 36 | 13 | 37 | 14 | | 333 | 16 | 333 | 16 | |

© 2019 Ma SJ et al. *JAMA Network Open.*

| | | | | | | | | | | |
|---|---|---|---|---|---|---|---|---|---|---|
| | | | | | | | | | | |
| Unplanned readmission within 30 days | | | | | 0.49 | | | | | 0.76 |
| No | 244 | 91 | 236 | 88 | | 1814 | 89 | 1815 | 89 | |
| Yes | 10 | 4 | 9 | 3 | | 119 | 6 | 108 | 5 | |
| Others | 4 | 1 | 6 | 2 | | 30 | 1 | 36 | 2 | |
| NA | 10 | 4 | 17 | 6 | | 79 | 4 | 83 | 4 | |

N: number or count; NA: not available or unknown; IQR: interquartile range; diff: differentiated; ref: reference; dx: diagnosis

© 2019 Ma SJ et al. *JAMA Network Open.*
